# People Living With Dementia in Rural Areas are Most Likely to Report Unmet Service Needs in Home‐ and Community‐Based Services in the United States

**DOI:** 10.1111/jrh.70230

**Published:** 2026-10-23

**Authors:** Romil R. Parikh, Eric Jutkowitz, Jack M. Wolf, Benjamin W. Langworthy, Chanee D. Fabius, Stephanie Giordano, Tetyana P. Shippee

**Affiliations:** ^1^ Evidence‐Based Practice & Impact Center, Division of Nephrology and Hypertension, University of Kansas Medical Center Kansas City Kansas USA; ^2^ Division of Health Policy & Management University of Minnesota School of Public Health Minneapolis Minnesota USA; ^3^ Department of Biostatistics and Epidemiology University of Pennsylvania Perelman School of Medicine Philadelphia Pennsylvania USA; ^4^ Division of Biostatistics and Health Data Science University of Minnesota School of Public Health Minneapolis Minnesota USA; ^5^ Center on Aging, University of Connecticut School of Social Work Hartford Connecticut USA; ^6^ Division of Health Policy & Management Johns Hopkins Bloomberg School of Public Health Baltimore Maryland USA; ^7^ Human Services Research Institute Cambridge Massachusetts USA

**Keywords:** community‐based care, community‐based services, dementia care, dementia‐capable systems, Medicaid HCBS, long‐term care, LTSS quality, rural disparities, rural health

## Abstract

**Purpose:**

Most older adults prefer aging in place, for which home‐ and community‐based services (HCBS) play an essential role. However, it is unknown whether rural‐urban differences in unmet HCBS needs differ by dementia status, which is an important evidence gap to develop targeted interventions to improve HCBS access. Therefore, we evaluated associations of rural‐urban continuum (RUCA classification) with consumer‐reported unmet HCBS needs by dementia status.

**Methods:**

We analyzed data from the National Core Indicators‐ Aging & Disability survey (2016‐2019; 14,645 community‐dwelling consumers; age, ≥65years; 23 states) using logistic regression, comparing adjusted odds ratios (OR) of consumer‐reported unmet HCBS needs across rural‐urban continuum, stratified by dementia.

**Findings:**

Among people living with dementia (PLWD, n = 2438), compared to metropolitan areas, prevalence of unmet HCBS needs was greater in micropolitan areas (35.4% versus 30.7% consumers; OR, 1.32, 95% CI, 0.98–1.77), small towns (36.3% consumers; OR, 1.44, 95% CI, 0.97–2.13), and rural areas (44.8% consumers; OR, 1.83, 95% CI, 1.11–3.04). These associations were reversed in those without dementia (n = 12,207). Compared with metropolitan areas, prevalence of unmet HCBS needs was lower among those in micropolitan areas (28.7% vs 31.4% consumers; OR, 0.82; 95% CI 0.72–0.93), small towns (29.3%; OR 0.85; 95% CI 0.73–1.00) and rural areas (25.4%; OR, 0.73, 95% CI, 0.58‐0.90).

**Conclusion:**

Among PLWD, unmet HCBS needs are significantly greater in rural areas. These findings highlight the critical need for dementia‐capable HCBS in rural areas to improve equitable access to these essential services among PLWD.

## 1 Introduction

Home‐ and community‐based services (HCBS) are a cornerstone of long‐term services and supports (LTSS) in the United States, enabling older adults with functional or cognitive impairments to remain in their homes and communities rather than enter institutional care [1, 2]. Over the past decade, Medicaid rebalancing initiatives have accelerated a nationwide shift toward HCBS as the preferred mode of LTSS delivery, mirroring strong consumer preferences for ‘aging‐in‐place’ [3, 4]. Yet, despite growing reliance on HCBS, substantial gaps persist in access to needed services, especially among populations at elevated risk of functional dependence and chronic conditions [2, 5, 6].

Older adults living with dementia face disproportionately high care needs due to progressive cognitive decline, behavioral symptoms, and increasing reliance on both paid and unpaid support [7, 8]. Prior research indicates higher levels of unmet need for HCBS among community‐dwelling people with dementia, potentially leading to poorer health and community‐living outcomes [2, 9, 10]. Rural communities experience persistent barriers to accessing HCBS, which may amplify the risk of unmet HCBS needs for rural older adults with dementia, whose care needs are more intensive and complex [5, 6, 7, 8, 9]. Despite a well‐established literature on rural–urban disparities in healthcare access and on the elevated support needs associated with dementia, few studies have examined how the intersection of rurality and dementia shapes consumer‐reported unmet needs within publicly funded HCBS programs. Understanding this intersection is increasingly critical as states face widening workforce shortages, expanding dementia prevalence, and growing demand for equitable, community‐based LTSS [2, 8].

Using a large, multi‐state sample of HCBS consumers, this manuscript evaluates the research question: are there any patterns of rural–urban differences in unmet service needs by dementia status? Identifying such patterns can inform targeted policy responses, guide resource allocation, and support dementia‐capable HCBS reforms aimed at ensuring that all older adults, regardless of dementia status or place of residence, receive the support they need to successfully age in place.

## 2 Methods

Our study was reviewed and approved by the Institutional Review Board at the [Redacted for Blinding]. We utilized data from the National Core Indicators Aging and Disability (NCI‐AD) survey.

### 2.1 Data Sources

The NCI‐AD survey is a comprehensive evaluation of individuals receiving publicly funded LTSS, incorporating measures to evaluate their quality of life, care experiences, satisfaction with care, health outcomes, and the performance of LTSS [2, 10]. Performance measures encompass aspects such as service coordination, access, choice, and safety. Proxies are utilized for participants unable to directly engage in the survey. The NCI‐AD survey is jointly administered by the ADvancing States, the Human Services Research Institute (HSRI), and various state Medicaid, aging, and disability agencies. Participation in the survey is voluntary, with states selecting which waiver programs to sample from (more details in supplemental methods).

### 2.2 Study Population

For this study, data from three consecutive annual waves spanning from 2016 to 2019 were combined to achieve a robust sample size. Previously, we conducted a sensitivity analysis to compare estimates of HCBS use and consumer‐reported unmet service needs from individual waves with those from the pooled sample, revealing no significant differences in results [2]. Over the specified period, a total of 24,348 consumers aged 65 and above participated in the NCI‐AD survey. We excluded all participants (n = 233) from one state with a very high level of missing data (>75% missing data on most covariates). We restricted our study to community‐dwelling older adults, defined as those living in their own or family homes, senior living apartments, group homes, adult family homes, foster homes, or host homes. Consumers residing in assisted living, nursing homes, or continuing care retirement communities were excluded (n = 7,749 consumers). These residential settings represent care environments in which service delivery, staffing, and care coordination differ substantially from community‐dwelling HCBS recipients. Because the study objective was to evaluate unmet HCBS needs among community‐dwelling older adults, we excluded individuals residing in institutional and residential care settings to maintain a conceptually similar study population. Further exclusions were made for 1,989 consumers whose dementia status remained missing after the NCI‐AD ascertainment process, which incorporates both state administrative records and, when administrative records do not indicate a dementia diagnosis, a standardized survey question regarding a prior diagnosis of Alzheimer's disease or another dementia. Consequently, the final analytic sample [Supplemental Figure 1] comprised 14,645 community‐dwelling older‐adult HCBS consumers.

### 2.3 Measurement of Unmet HCBS Needs

We identified consumers with self‐reported unmet HCBS needs by examining responses to the survey question in the consumer‐reported section, “Do the long‐term care services you receive meet your current needs and goals?” [2, 9]. The responses to the question included yes, some, or no. Participants who indicated inadequacy of their current services in meeting their needs (responses “no” or “some”) were categorized as having unmet needs.

### 2.4 Ascertainment of Rural‐Urban Classification of Residence

Rural‐urban continuum was measured using the Rural–Urban Commuting Area (RUCA) taxonomy, a ZIP code‐based classification system [11]. RUCA codes incorporate both population density and commuting flows, allowing for a nuanced characterization of the rural–urban continuum beyond simple population size. Information about the RUCA‐based residence location is captured in the Background Information section of the NCI‐AD adult consumer survey, from administrative records such as the Medicaid Management Information System. The variable was categorized into four levels: (1) Metropolitan, representing urban cores with populations ≥50,000 and surrounding areas with substantial commuting to these metropolitan centers; (2) Micropolitan, representing urban clusters of 10,000‐49,999 residents and surrounding areas with substantial commuting to these centers; (3) Small Town, representing urban clusters of fewer than 10,000 residents and surrounding areas with substantial commuting to these communities; and (4) Rural, representing areas outside urbanized areas and small towns where the primary commuting flow is to other rural areas [11].

### 2.5 Dementia Ascertainment

In this study, we ascertained whether an individual had received a diagnosis of dementia (yes or no) using the Background Information section (details in supplemental methods) of the NCI‐AD survey [12]. In the Background Information section, a diagnosis of dementia is ascertained from state administrative records such as the Medicaid Management Information System [12]. In instances where a dementia diagnosis is absent from state administrative records, surveyors pose the following question to consumers: “Has the individual been informed of a diagnosis of Alzheimer's disease or any other form of dementia (manifesting as a decline in memory or other cognitive abilities that may impede everyday functioning)?” Subsequently, consumers who answer affirmatively are classified as possessing dementia, whereas those responding negatively are categorized as not having dementia [12].

### 2.6 Measurement of Covariates

Covariates were selected based on previously published literature that aligns with Andersons Behavioral Model for health service use [2, 10, 12, 13, 14]. We included demographic covariates including age, sex (male/female), race (Asian, Black, Hispanic/Latino, white, or other/multiracial), marital status (Single; Married or has a domestic partner; Separated or divorced; and Widowed), residence (own/ family home, senior living, or other congregate setting like group homes), median ZIP code income (dichotomized at USD 30,000), funding program category (Program of All‐inclusive Care for the Elderly [PACE], Older Americans Act, or Medicaid), enrollment in Medicare, self‐ versus proxy‐respondent, and whether the consumer has an appointed legal guardian. We included indicators of food insecurity such as skipping meals due to finances and access to healthy food (yes/often versus no). We included indicators of functional status using responses to the following three questions: (1) “How much assistance with everyday activities do you generally need? Things like preparing meals, housework, shopping or taking your medications”; and (2) “How much assistance with self‐care do you generally need? Things like bathing, dressing, going to the bathroom, eating or moving around your home/ where you live” (both coded as none versus some/a lot); and (3) use or reported need for scooter, walker, or wheelchair (yes versus no). Health‐related covariates included overall self‐rated health, measured on a 5‐point scale from poor to excellent, and self‐rated vision and hearing. Disability‐related covariates included developmental disability, physical disability, and brain injury. We also included diagnosed mental health conditions, including bipolar disorder, schizophrenia, and major depressive disorder, as documented in the background information section or during survey administration.

### 2.7 Statistical Analysis

We summarized participant characteristics overall and stratified by dementia diagnosis as percentage or median (IQR). We estimated the prevalence of unmet HCBS needs stratified by rurality and dementia. We estimated the conditional probability of reporting unmet needs as a function of RUCA and all covariates stratified by dementia diagnosis using logistic regression models. We tested a difference in RUCA's association with unmet HCBS needs between people living with and without dementia by performing a Chow‐like Wald test to compare the set of adjusted log odds ratios associated with the rural‐urban continuum variable between the dementia and no dementia models. We reported point estimates and 95% confidence intervals for the adjusted odds ratios for both models and the chi‐squared statistic and p‐value from the test of equality of associations. All test results were considered statistically significant if p<0.05. Missing data was 15.6% for having a legal guardian, 9.2% for median income, and <5% for other covariates. We used multiple imputation by chained equations to address missing data. All results were pooled across 10 imputed datasets. All analyses were performed in R v4.1.3.

## 3 Results

Among 14,645 community‐dwelling older adults receiving publicly funded HCBS, 2438 (17%) had a dementia diagnosis. Individuals with dementia were older (median 81 vs. 76 years) and more likely to be married or widowed, reside in metropolitan areas, and be enrolled in Medicaid HCBS waivers; they more frequently had a developmental disability, brain injury, and mental health conditions; yet, they were more likely to rate their health as very good or excellent, but reported poorer hearing ability (Table 1). Marked differences were observed in functional status: 71% of those with dementia required “a lot” of assistance with daily activities compared with 42% without dementia, and 58% required substantial self‐care assistance compared with 29%. Proxy respondents were more common among the dementia group.

**TABLE 1:** Characteristics of older adult, community dwelling respondents to the NCI‐AD 2016–19 by dementia diagnosis.

Characteristic	Overall N = 14,645	Dementia N = 2,438	No Dementia N = 12,207
Age (Years; Median (Q1, Q3))	77 (70, 84)	81 (74, 87)	76 (70, 83)
Female (vs not Female)	72%	72%	72%
Race/Ethnicity			
White	60%	55%	61%
Asian	3.3%	5.5%	2.9%
Black	23%	21%	24%
Hispanic or Latino	9.3%	14%	8.4%
Other/Multiracial	4.2%	4.8%	4.0%
ZIP Code RUCA Classification			
Metropolitan	72%	77%	71%
Micropolitan	15%	12%	16%
Small town	8.2%	6.9%	8.4%
Rural	4.7%	3.5%	4.9%
Marital Status			
Single	14%	12%	14%
Married/Domestic Partner	21%	24%	20%
Separated/Divorced	26%	20%	27%
Widowed	40%	44%	39%
Residence Setting			
Own or family house or apartment	89%	86%	89%
Group home, adult family home, foster home, or host home	3.6%	8.1%	2.7%
Senior living	7.7%	5.9%	8.0%
Funding Program			
Medicaid	70%	80%	68%
OAA	25%	14%	27%
PACE	4.8%	5.8%	4.6%
Medicare Enrollee	91%	92%	91%
Have Legal Guardian	10%	24%	7.3%
ZIP Code Median Income ≥ $30,000	88%	90%	88%
Physical Disability	56%	50%	57%
Developmental Disability	7.2%	10%	6.5%
Brain Injury	10%	15%	9.2%
Mental Health Condition	20%	25%	19%
*Consumers’ Self‐Reported Health and Functional Status*			
Overall Self‐Rated Health			
Poor	18%	20%	18%
Fair	39%	35%	40%
Good	30%	31%	30%
Very Good	9.8%	12%	9.4%
Excellent	2.4%	3.4%	2.2%
Self‐Described Hearing Ability			
Poor	17%	23%	16%
Fair	33%	31%	33%
Very good	50%	46%	51%
Self‐Described Vision Ability			
Poor	20%	21%	20%
Fair	39%	38%	39%
Very good	41%	42%	41%
Use or Reported Need of Scooter, Walker, or Wheelchair	84%	84%	85%
Residence Setting			
Own or family house or apartment	89%	86%	89%
Group home, adult family home, foster home, or host home	3.6%	8.1%	2.7%
Senior living	7.7%	5.9%	8.0%
Required Assistance for Daily Activities (e.g., meal prep, housework, shopping, etc.)			
None	10%	3.6%	11%
Some	43%	26%	47%
A lot	46%	71%	42%
Required Assistance for Self‐Care (e.g., bathing, dressing, eating, etc.)			
None	29%	14%	32%
Some	37%	28%	39%
A lot	33%	58%	29%
Proxy	13%	42%	6.9%

Values are % unless otherwise indicated and are averaged across multiple imputations. OAA = Older Americans Act; PACE = Program of All‐inclusive Care for the Elderly; Q1 = first quartile; Q3 = third quartile; RUCA = rural‐urban commuting area classification.

Across the full sample (Table 2), unmet needs were reported by 32.1% of HCBS consumers with dementia and 30.5% without dementia. Rural residence was consistently associated with greater likelihood of unmet needs among people living with dementia; in contrast, rurality exhibited a protective trend of decreasing unmet needs for HCBS consumers without dementia. Among individuals with dementia, unmet need increased steadily with rurality, ranging from 30.7% in metropolitan areas to 44.8% in rural areas‐ a 14.1 percentage‐point absolute increase. In contrast, a reverse relationship was observed among those without dementia; the prevalence of unmet needs decreased by 6% across the rural‐urban continuum, from 31.4% in metropolitan areas to 25.4% in rural areas.

**TABLE 2:** Prevalence of Consumer‐Reported Unmet Service Needs in Home‐ and Community‐Based Services (2016‐2019); Across the Rural‐Urban Continuum; Stratified by Dementia Status

	Dementia		No Dementia	
Area	Total Number of Consumers	**%** Reporting Unmet HCBS Needs (95% CI)	Total Number of Consumers	**%** Reporting Unmet HCBS Needs (95% CI)
Overall	2,438	32.1 (30.3, 34.0)	12,207	30.5 (29.6, 31.3)
Metropolitan	1,877	30.7 (28.6, 32.8)	8,667	31.4 (30.4, 32.4)
Micropolitan	293	35.4 (29.9, 41.0)	1,953	28.7 (26.6, 30.7)
Small Town	170	36.3 (28.9, 43.8)	977	29.3 (26.5, 32.1)
Rural	98	44.8 (33.9, 55.7)	610	25.4 (21.8, 29.0)

CI‐ confidence interval; HCBS‐ home‐ and community‐based services. The table reports unadjusted % prevalence in the sample.

Figure 1 (and Supplemental Table 1) shows that the adjusted association between rural‐urban residence and consumer‐reported unmet HCBS needs differed markedly by dementia status. The overall Chow‐like test indicated at least one difference in adjusted log odds ratios by dementia diagnosis (p<0.001). Among older adults living with dementia, residence outside metropolitan areas was associated with higher odds of unmet needs, with the largest increase observed in rural areas. Compared with metropolitan residents, rural residents with dementia had 83% higher adjusted odds of reporting unmet HCBS needs (Figure 1). Older adults with dementia living in micropolitan and small‐town areas also had 32% and 44% higher adjusted odds of unmet needs, respectively (Figure 1); although these associations did not reach statistical significance. In contrast, among older adults without dementia, rural residence was associated with lower odds of unmet needs. Compared with metropolitan residents, those living in micropolitan, small‐town, and rural areas had 18%, 15%, and 27% lower adjusted odds, respectively, of reporting unmet HCBS needs (Figure 1).

**FIGURE 1:**
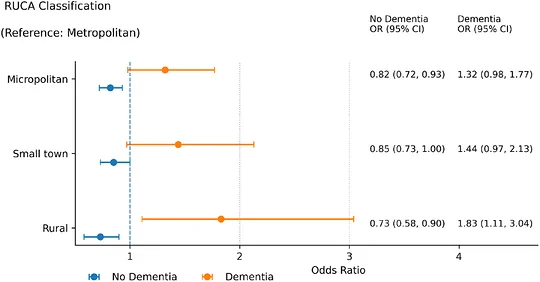
Associations of Rural‐Urban Continuum With Consumer‐Reported Unmet Service Needs in Home‐ and Community‐Based Services (2016‐2019); Stratified by Dementia Status. Reference group = metropolitan areas. Models are adjusted for all covariates in Table 1. Chow‐like test p‐value is <0.001, indicating that the Odds Ratios for people with dementia are significantly different from the odds ratios for people without dementia. RUCA = ZIP code‐based rural‐urban commuting area codes; OR = odds ratios; CI = confidence interval.

## 4 Discussion

In this multi‐state analysis of publicly funded HCBS consumers, we observed a pronounced divergence in how rural–urban residence relates to consumer‐reported unmet HCBS needs depending on dementia status. Among older adults living with dementia, residence in rural areas was associated with substantially higher odds of unmet HCBS needs compared with metropolitan areas. In contrast, among those without dementia, rurality was associated with *lower* unmet HCBS needs, an unexpected pattern that contrasts with the elevated risks observed for people with dementia. These findings may suggest that dementia can shift how place‐based resources and constraints translate into service adequacy.

Our results build on prior work documenting heightened vulnerability among community‐dwelling individuals with dementia, who typically require more intensive, continuous, and cognitively tailored support [6, 7, 8]. Extensive literature has shown that people with dementia experience greater unmet need for assistance with daily activities, behavioral management, care coordination, and supervision [8, 15, 16, 17, 18]. These needs, when unaddressed, can potentially lead to preventable hospitalizations, accelerated functional decline, and early institutionalization [19, 20, 21, 22, 23, 24, 25]. At the same time, rural LTSS systems continue to face persistent workforce shortages, limited dementia‐specific training, long travel distances for in‐home service delivery, and fewer respite and caregiver‐support programs [6, 26, 27, 28, 29]. Collectively, these structural barriers likely produce the amplified unmet needs observed among rural HCBS consumers with dementia in our study [6].

The inverse association among older adults without known dementia is more difficult to reconcile with established rural–urban disparities in healthcare access. This finding may reflect several mechanisms. First, non‐dementia HCBS consumers in rural areas may have less complex support needs that can be met through available personal care or chore services despite workforce limitations [30]. Second, rural communities may benefit from stronger informal caregiving networks that help buffer unmet service needs [31]. Although informal caregivers play an essential role for older adults both with and without dementia, individuals without cognitive impairment may have less complex care needs that can be adequately supplemented by family or community support. In contrast, people living with dementia often require continuous supervision, behavioral management, care coordination, and dementia‐specific services that may exceed the capacity of informal caregiving alone [8]. This may increase reliance on formal dementia‐capable HCBS, which may not be readily available in rural areas [31]. Third, our measure of unmet HCBS needs (capturing self‐reported gaps) may operate differently for people with preserved cognition, who may be better positioned to navigate local service systems or adapt expectations to available resources. These possibilities underscore that rurality does not uniformly confer disadvantage; rather, its impact depends on the intersection of place with dementia status and support requirements [32].

The implications for research, policy, and practice are substantial. Future work should delineate which service domains drive the excess unmet needs among rural adults with dementia and examine how caregiver availability, workforce capacity, and organizational models of HCBS delivery jointly shape these outcomes [2, 6, 8, 13, 26, 33, 34]. Policymakers may consider targeted enhancements to rural HCBS infrastructures such as dementia‐specific workforce development, expanded care coordination, and reimbursement models that account for travel time and service intensity. Clinically, care coordinators and HCBS agencies may need to actively screen for unmet needs among rural clients with dementia and implement proactive, dementia‐capable care planning.

We acknowledge several constraints within our study. Firstly, we conducted secondary data analysis of cross‐sectional survey design; so, we were unable to apply a rigorous causal study design. Secondly, we relied on administrative records and self‐report for dementia diagnosis with potential for misclassification [12]. However, these data reflect real‐world systems and consumers, yielding pragmatically applicable insights. Third, interpretation of the NCI‐AD survey data must consider the inherent limitations of the survey methods and procedures. Participation in the NCI‐AD survey by states is voluntary, and the level of state engagement varies across different survey years. While NCI‐AD imposes standardized criteria for state samples, including mandatory thresholds for sample sizes and margins of error, states customize their sampling strategies to address specific concerns. These factors hinder extrapolation of findings to non‐participating states. Fourth, biases stemming from unmeasured and residual confounding may persist in our observational study; for example, presence of a live‐in caregiver was not accounted for. Fifth, while we compare rural to urban communities, we were unable to evaluate regional or state‐level heterogeneity in rural and urban HCBS access. Rural communities differ considerably in geography, healthcare infrastructure, Medicaid HCBS policies, and workforce availability, and these contextual factors may modify the relationship between rural residence and unmet HCBS needs [33]. Future studies using larger samples or nationally representative datasets should examine whether these associations differ across regions and identify region‐specific barriers to dementia‐capable HCBS delivery. The sample also included relatively few participants living in group homes or adult foster care, limiting our ability to assess whether associations differed by living arrangement or by living alone versus with others. Finally, our analyses used data collected between 2016 and 2019, before the COVID‐19 pandemic substantially altered HCBS delivery nationwide [9]. Future studies should leverage more recent data to investigate if these associations persisted after the pandemic.

## 5 Conclusion

In this multi‐state study of community‐dwelling older HCBS consumers, dementia status substantially modified the association between rural–urban residence and unmet service needs. Rurality was linked to markedly higher unmet needs among individuals with dementia, but unexpectedly lower unmet needs among those without dementia. These findings highlight the critical intersection of dementia and geography in shaping HCBS adequacy. Building dementia‐capable, place‐responsive care systems will be essential to supporting equitable opportunities for aging in place across the rural–urban continuum.

## Funding

The research reported in this publication was supported by the National Institute on Aging of the National Institutes of Health under Award Numbers RF1AG069771 and R01AG069771S1.

## Ethics Approval, Consent to Participate and Data Availability

This secondary analysis was approved by the Institutional Review Board of University of Minnesota under protocol STUDY00010799. This study is also covered by data use agreements with the Minnesota Department of Human Services (42031D) and Human Services Research Institute (39008D). We used data from the National Core Indicators‐Aging and Disability survey and Minnesota Department of Human Services. Investigators can contact the corresponding author for information about how to apply for access to the data used in our analyses.

## Conflicts of Interest

Dr. Jutkowitz is a co‐founder and on the board of directors of Plans4Care Inc. a digital health company that provides personalized dementia care on‐demand. He also serves on the finance committee for PACE‐RI. No other authors report conflicts of interest.

## Supporting information




**Supporting File**: jrh70230‐sup‐0001‐SuppMat.docx
