# Supplementary material for: People Living With Dementia in Rural Areas are Most Likely to Report Unmet Service Needs in Home‐ and Community‐Based Services in the United States
Source: J Rural Health. 2026 Oct 23;42(4):e70230. doi: 10.1111/jrh.70230 (PMC13692696; doi:10.1111/jrh.70230)
Supplement: Supplementary file 1 [file JRH-42-0-s001.docx]

**Online Supplement**

**Supplemental Methods:**

States utilize a probabilistic sampling method to select participants from the eligible population for their services, aiming to achieve a maximum margin of error of 5% at a 95% confidence level. Prior to conducting interviews, detailed background information about survey participants, covering demographics, personal characteristics, legal status, services received, and clinical diagnoses, is systematically collected from administrative records and then linked to survey responses. This linkage creates a comprehensive data record for each participant, combining both administrative and survey data. Data from the survey's Background Information section, including race, gender, mental health diagnoses, Alzheimer's disease and related dementias, and disabilities, is typically obtained through state administrative channels such as case management, managed care, and Medicaid billing records. Administrative records, rather than self-reporting, are crucial for determining service types, often sourced from the Medicaid Management Information System.

The process of sample selection typically begins within 3-4 months of survey initiation. However, due to the extended data collection timeline, individuals may be surveyed for up to 8 months after sample selection. The National Core Indicators for Aging and Disabilities (NCI-AD) background information section relies on state administrative records for demographic and service-related data. In cases where relevant administrative records are unavailable, individuals may be asked directly at the end of the survey. However, certain subsections of the Background Information section, such as primary long-term services and supports (LTSS) funding source, primary program, services received through the program, duration of participation, self-directed support, and legal guardianship status, require data collection from administrative records. To be eligible for the survey, individuals must currently be receiving at least one active service (e.g., personal care, homemaker, transportation) at least twice a week for approximately 3 months. Active service receipt is a prerequisite for survey participation.

**Statistical Supplement:**

Differences in the associations between RUCA unmet need between those living with and without ADRD were evaluated using a Wald test. Letting $\hat{\beta}_{\boldsymbol{RUCA, ADRD}}$ and $\hat{\beta}_{RUCA, No ADRD}$ be the respective vectors of maximum likelihood estimates of the log odds ratios for all RUCA levels versus the reference category of metropolitan residence within the ADRD and No ADRD strata, we tested the null hypothesis that $\beta_{RUCA,ADRD}=\beta_{RUCA, No ADRD}$ via the test statistic

$(\hat{\beta}_{RUCA, ADRD}-\hat{\beta}_{RUCA, ADRD})^{T}({\hat{V}(\hat{\beta}_{RUCA, ADRD}) +\hat{V}(\hat{\beta}_{RUCA, No ADRD}) )}^{-1}(\hat{\beta}_{RUCA, ADRD}-\hat{\beta}_{RUCA, ADRD})$

which follows an asymptotic $\chi^{2}$ distribution with 3degrees of freedom under the null hypothesis, and $\hat{V}(\hat{\beta}_{RUCA, ADRD})$ and $\hat{V}(\hat{\beta}_{RUCA, No ADRD})$ are the estimated variances of the respective maximum likelihood estimators.

**Covariate Selection Rationale**

We selected covariates a priori using Andersen's Behavioral Model of Health Service Use, which proposes that service use and unmet needs are shaped by predisposing characteristics, enabling resources, and need-related factors.^2^ Rural-urban residence, measured using the Rural-Urban Commuting Area classification, was the primary exposure.^11^ Predisposing characteristics included age, sex, race and ethnicity, marital status, and dementia status. ^2^ Enabling factors represented resources and access to care, including residential setting (own/family home, senior living, or other community-based congregate setting), Medicaid HCBS funding program (Medicaid, Older Americans Act, or Program of All-Inclusive Care for the Elderly), Medicare enrollment, ZIP code median income, legal guardianship, proxy respondent status, and indicators of food insecurity.^2^ Need factors reflected the individual's health status and care requirements, including self-rated health, hearing and vision, functional assistance needs for activities of daily living and self-care, use or need for mobility devices, and diagnoses of physical disability, developmental disability, brain injury, and mental health conditions.^2^ Guided by this conceptual framework, these variables were included as potential confounders in multivariable analyses examining the association between rural-urban residence and consumer-reported unmet HCBS needs. To improve internal validity and maintain a clinically comparable population, analyses were restricted to community-dwelling adults aged 65 years or older receiving publicly funded HCBS.

**Supplemental Tables**

**Supplemental Table 1.** Odds ratios of overall unmet need conditional on covariates for adult HCBS consumers with and without ADRD. Chow-like tests of equality of adjusted effects between those with and without ADRD were performed and reported for each covariates. Results were pooled across multiple imputations.

|  | ADRD | No ADRD | Comparison |
| --- | --- | --- | --- |
| **Characteristic** | OR (95% CI)^12^ | OR (95% CI)^12^ | $\chi^{2}$, DF, p-value^13^ |
| ZIP Code RUCA Classification |  |  | 18.3, 4, 0.001** |
| Metropolitan | — | — |  |
| Micropolitan | 1.32 (0.98, 1.77) | 0.82 (0.72, 0.93)** |  |
| Small town | 1.44 (0.97, 2.13) | 0.85 (0.73, 1.00) |  |
| Rural | 1.83 (1.11, 3.04)* | 0.73 (0.58, 0.90)** |  |
| Age (Years) | 0.99 (0.97, 1.00)* | 0.99 (0.98, 1.00)** | 0.5, 1, 0.49 |
| Female (vs not Female) | 0.86 (0.69, 1.07) | 1.05 (0.95, 1.17) | 2.8, 1, 0.094 |
| Marital Status |  |  | 7.0, 3, 0.071 |
| Single | — | — |  |
| Married/Domestic Partner | 0.94 (0.66, 1.34) | 0.99 (0.85, 1.15) |  |
| Separated/Divorced | 0.88 (0.61, 1.27) | 1.19 (1.03, 1.37)* |  |
| Widowed | 1.14 (0.81, 1.59) | 1.04 (0.90, 1.20) |  |
| Race/Ethnicity |  |  | 13.9, 4, 0.008** |
| White | — | — |  |
| Asian | 1.21 (0.78, 1.88) | 1.41 (1.09, 1.83)** |  |
| Black or African-American | 1.52 (1.16, 1.98)** | 1.00 (0.89, 1.12) |  |
| Hispanic or Latino | 1.53 (1.07, 2.18)* | 0.89 (0.73, 1.08) |  |
| Other/Multiracial | 1.04 (0.64, 1.68) | 0.96 (0.77, 1.20) |  |
| Waiver |  |  | 4.3, 2, 0.12 |
| Medicaid | — | — |  |
| OAA | 1.26 (0.87, 1.84) | 1.92 (1.67, 2.21)*** |  |
| PACE | 0.62 (0.38, 1.02) | 0.61 (0.47, 0.79)*** |  |
| Medicare Enrollee | 1.31 (0.90, 1.91) | 1.14 (0.97, 1.34) | 0.4, 1, 0.50 |
| Have Legal Guardian | 0.98 (0.77, 1.25) | 0.95 (0.80, 1.14) | 0.0, 1, 0.87 |
| Overall Health |  |  | 7.7, 4, 0.10 |
| Poor | — | — |  |
| Fair | 0.84 (0.65, 1.09) | 0.82 (0.73, 0.92)*** |  |
| Good | 0.56 (0.42, 0.74)*** | 0.71 (0.62, 0.81)*** |  |
| Very Good | 0.44 (0.30, 0.65)*** | 0.66 (0.55, 0.79)*** |  |
| Excellent | 0.54 (0.30, 0.98)* | 0.47 (0.33, 0.67)*** |  |
| Physical Disability | 0.96 (0.77, 1.20) | 1.16 (1.05, 1.28)** | 2.3, 1, 0.13 |
| Developmental Disability | 1.06 (0.69, 1.62) | 0.92 (0.73, 1.15) | 0.3, 1, 0.56 |
| Brain Injury | 0.99 (0.70, 1.38) | 1.17 (0.97, 1.42) | 0.8, 1, 0.38 |
| Mental Health Condition | 1.32 (1.06, 1.65)* | 1.26 (1.13, 1.40)*** | 0.2, 1, 0.68 |
| Skip Meal Due to Finances | 1.59 (1.06, 2.40)* | 1.82 (1.59, 2.08)*** | 0.4, 1, 0.55 |
| Access to Healthy Food | 0.34 (0.25, 0.47)*** | 0.41 (0.36, 0.45)*** | 0.9, 1, 0.33 |
| Self Described Hearing Ability |  |  | 8.6, 2, 0.014* |
| Poor | — | — |  |
| Fair | 0.75 (0.58, 0.97)* | 0.89 (0.79, 1.01) |  |
| Very good | 0.98 (0.75, 1.27) | 0.81 (0.71, 0.92)*** |  |
| Self Described Vision Ability |  |  | 4.2, 2, 0.12 |
| Poor | — | — |  |
| Fair | 1.00 (0.77, 1.29) | 0.76 (0.68, 0.86)*** |  |
| Very good | 0.89 (0.68, 1.17) | 0.68 (0.60, 0.76)*** |  |
| Use or Reported Need of Scooter, Walker, or Wheelchair | 1.14 (0.86, 1.50) | 1.07 (0.94, 1.21) | 0.2, 1, 0.69 |
| Residence Setting |  |  | 2.5, 2, 0.28 |
| Own or family house or apartment | — | — |  |
| Group home, adult family home, foster home, or host home | 0.57 (0.36, 0.90)* | 0.74 (0.54, 0.99)* |  |
| Senior living | 0.75 (0.50, 1.13) | 1.02 (0.87, 1.20) |  |
| ZIP Code Median Income ≥ $30,000 | 1.48 (1.03, 2.13)* | 1.22 (1.05, 1.40)** | 1.0, 1, 0.32 |
| Required Assistance for Daily Activities |  |  | 2.3, 2, 0.31 |
| None | — | — |  |
| Some | 0.83 (0.46, 1.51) | 1.26 (1.07, 1.48)** |  |
| A lot | 1.04 (0.56, 1.93) | 1.36 (1.12, 1.64)** |  |
| Required Assistance for Self Care |  |  | 3.4, 2, 0.18 |
| None | — | — |  |
| Some | 0.85 (0.60, 1.21) | 1.11 (0.99, 1.24) |  |
| A lot | 0.86 (0.59, 1.26) | 1.26 (1.09, 1.46)** |  |
| Proxy | 1.05 (0.84, 1.31) | 0.95 (0.80, 1.13) | 0.5, 1, 0.48 |
| ^1^*p<0.05; **p<0.01; ***p<0.001, ^2^CI = Confidence Interval, ^3^DF = Degrees of Freedom | | |  |

Supplemental Figure 1. Participant Selection Flow Diagram
